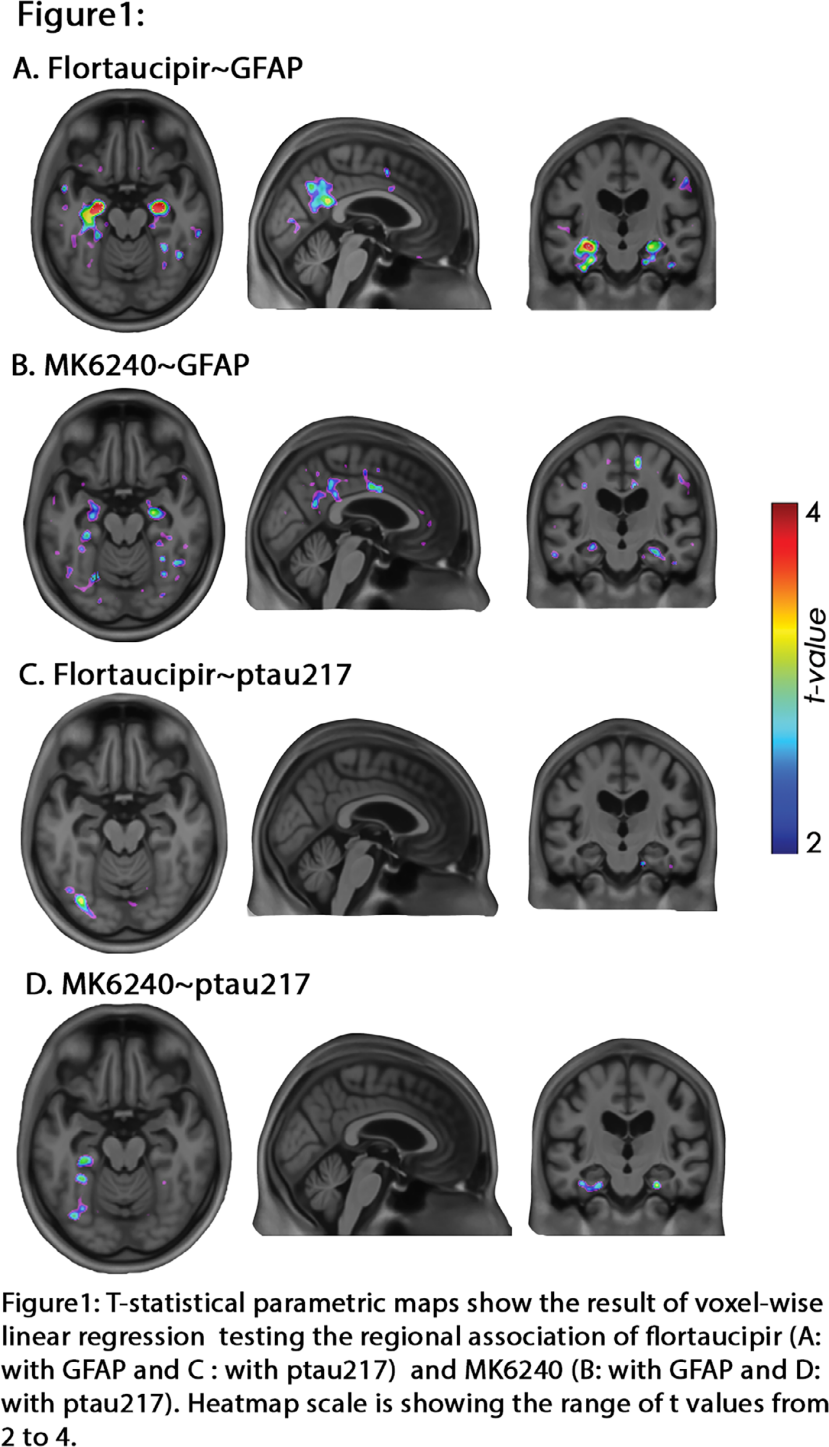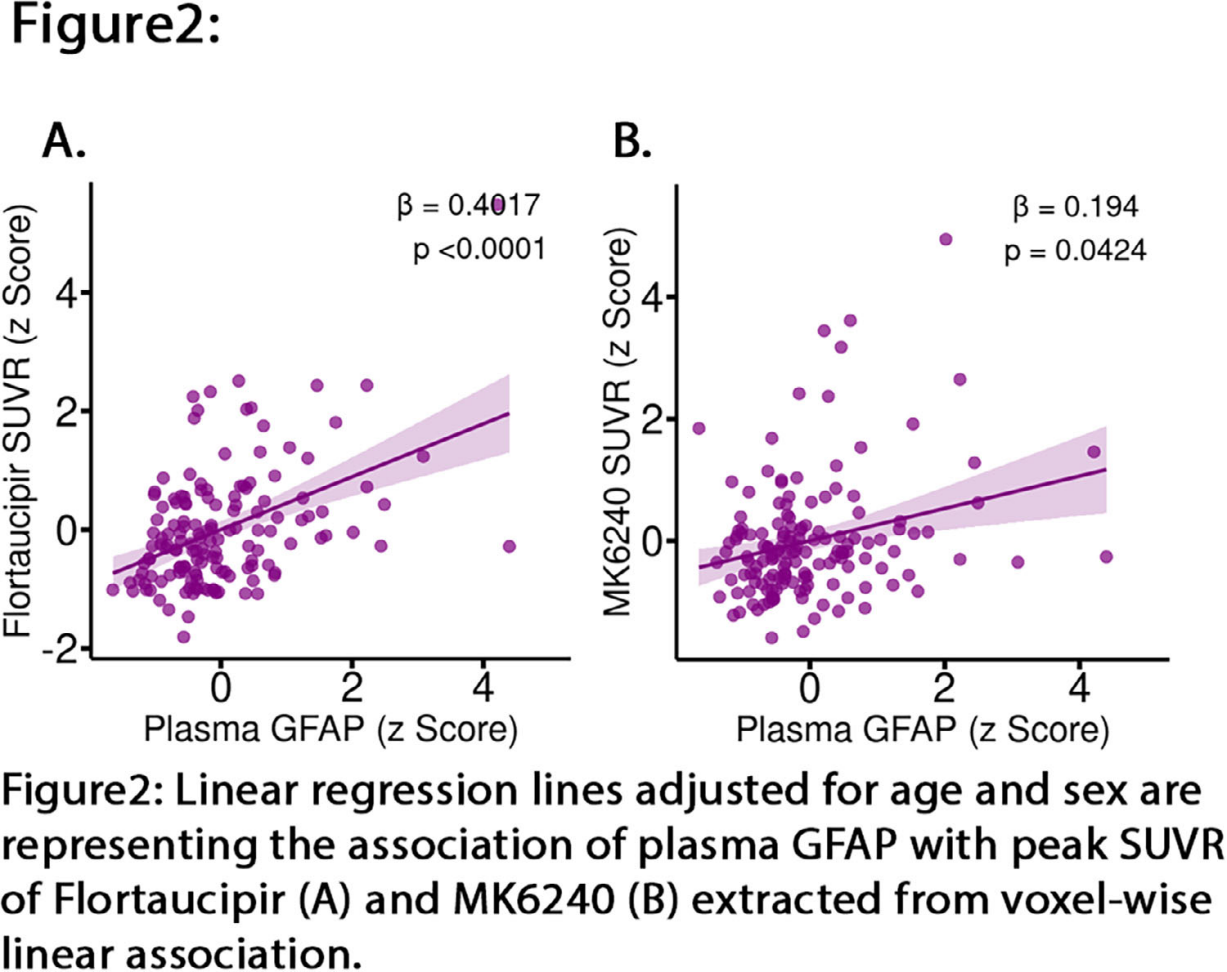# Tau‐PET and plasma GFAP association in cognitively unimpaired Aβ‐PET negative individuals

**DOI:** 10.1002/alz70856_105935

**Published:** 2026-01-07

**Authors:** Pampa Saha, Bruna Bellaver, Guilherme Povala, Pamela C.L. Ferreira, Guilherme Bauer‐Negrini, Livia Amaral, Firoza Z Lussier, Thomas K Karikari, Matheus Scarpatto Rodrigues, Markley Silva Oliveira, Andreia Rocha, Cynthia Felix, Emma Ruppert, Marina Scop Medeiros, Carolina Soares, Joseph C. Masdeu, Dana L Tudorascu, David N. Soleimani‐Meigooni, Juan Fortea, Val J Lowe, Hwamee Oh, Belen Pascual, Brian A. Gordon, Pedro Rosa‐Neto, Suzanne L. Baker, Tharick A Pascoal

**Affiliations:** ^1^ University of Pittsburgh, Pittsburgh, PA, USA; ^2^ Houston Methodist Research Institute, Houston, TX, USA; ^3^ Memory and Aging Center, Weill Institute for Neurosciences, University of California San Francisco, San Francisco, CA, USA; ^4^ Sant Pau Memory Unit, Department of Neurology, Hospital de la Santa Creu i Sant Pau, Institut d'Investigació Biomèdica Sant Pau (IIB SANT PAU), Facultad de Medicina ‐ Universitat Autònoma de Barcelona, Barcelona, Spain; ^5^ Mayo Clinic, Rochester, MN, USA; ^6^ Brown University, Providence, RI, USA; ^7^ Washington University in St. Louis, School of Medicine, St. Louis, MO, USA; ^8^ Translational Neuroimaging Laboratory, The McGill University Research Centre for Studies in Aging, Montréal, QC, Canada; ^9^ Lawrence Berkeley National Laboratory, Berkeley, CA, USA; ^10^ University of Pittsburgh School of Medicine, Pittsburgh, PA, USA

## Abstract

**Background:**

We showed that astrocyte reactivity, as measured by plasma GFAP levels, influences Aβ mediated tau PET pathology in cognitively unimpaired (CU) Aβ‐positive individuals. However, the link between GFAP and tau PET in individuals without detectable Aβ pathology remains elusive. The aim of the current study is to investigate the association between plasma GFAP and tau PET in CU Aβ PET‐negative individuals.

**Method:**

We studied 147 CU Aβ PET‐negative participants from the HEAD cohort with plasma GFAP and *p*‐tau217, as well as tau PET Flortaucipir and MK6240 data. Aβ positivity was determined by Aβ PET visual reading and Centiloid 12. Voxel‐wise linear regression models adjusted for age and sex tested the association of plasma GFAP and *p*‐tau217 with tau PET. Further, the associations of plasma GFAP with peak tau PET SUVR values extracted from voxel‐wise association were fitted with a linear regression model adjusted for age and sex.

**Result:**

Voxel‐wise analysis showed that plasma GFAP levels, but not plasma *p*‐tau217 levels, were associated with tau PET in the medial temporal lobe (e.g., amygdala, entorhinal cortex, hippocampus) predominantly for Flortaucipir tau PET [Figure 1A, B, C, D]. The association between plasma *p*‐tau217 and tau PET was weak in Aβ‐negative individuals. These results were similar when Aβ positivity was defined based on Centiloid 12. Furthermore, plasma GFAP and peak tau PET SUVR of Flortaucipir showed stronger association than that of MK6240 [Flortaucipir: β=0.4017, *p* <0.0001; MK6240: β=0.194, *p* = 0.0424; Figure 2A, B].

**Conclusion:**

We found an association between GFAP levels and tau PET uptake in individuals not expected to exhibit high levels of tau tangle‐related tracer uptake. Further analysis will be designed to elucidate the underpinning of this association, which could represent low levels of tau pathology, astrogliosis, or other factors.